# Central versus Peripheral CTEPH—Clinical and Hemodynamic Specifications

**DOI:** 10.3390/medicina58111538

**Published:** 2022-10-27

**Authors:** Monika Kaldararova, Iveta Simkova, Marcela Bohacekova, Adriana Reptova, Tereza Hlavata, Jozef Pacak, Jaroslav Lindner, Pavel Jansa

**Affiliations:** 1 Department of Cardiology and Angiology, CTEPH Expert Centre, Medical Faculty, The National Institute of Cardiovascular Diseases, Slovak Medical University, Pod Krasnou horkou 1, 833 48 Bratislava, Slovakia; 2 2nd Department of Surgery—Department of Cardiovascular Surgery, 1st Faculty of Medicine, General University Hospital, Charles University, U nemocnice 2, 128 02 Prague, Czech Republic; 3 Center for Pulmonary Hypertension, 1st Faculty of Medicine, General University Hospital, Charles University, U nemocnice 2, 128 02 Prague, Czech Republic

**Keywords:** chronic thromboembolic pulmonary hypertension (CTEPH), central CTEPH, peripheral CTEPH, risk factors, hemodynamic evaluation

## Abstract

*Background and Objectives:* Chronic thromboembolic pulmonary hypertension (CTEPH) is a chronic progressive disease, resulting from persistent arterial obstruction combined with small-vessel remodeling. Central and peripheral CTEPH are distinguished, according to the dominant lesion’s location. This is important for surgical or percutaneous interventional assessment or for medical treatment. *Material and Methods:* Eighty-one patients (51 male/30 female) with confirmed CTEPH were analyzed, while the CENTRAL type included 51 patients (63%) and the PERIPHERAL type 30 patients (37%). *Results:* A significant difference in CENTRAL type vs. PERIPHERAL type was determined in gender (male 72.5% vs. 46.7%; *p* = 0.0198). No difference was found in age, functional status, or echocardiographic parameters. Invasive hemodynamic parameters showed a significant difference in mean pulmonary arterial pressure (46 vs. 58 mmHg; *p* = 0.0002), transpulmonary gradient (34 vs. 47 mmHg; *p* = 0.0005), and cardiac index (2.04 vs. 2.5 L.min.m^2^; *p* = 0.02) but not in pulmonary vascular resistance. Risk factors showed a significant difference only in acute pulmonary embolism (93.8% vs. 60%; *p* = 0.0002) and malignancy (2% vs. 13.3%; *p* = 0.0426). *Conclusions:* Our study showed hemodynamic differences between CENTRAL type vs. PERIPHERAL type CTEPH with a worse hemodynamic picture in CENTRAL form. This may indicate a different pathophysiological response and/or possible additional influences contributing especially to the peripheral pulmonary bed affection.

## 1. Introduction

Chronic thromboembolic pulmonary hypertension (CTEPH) represents a specific group of pulmonary hypertension (PH), defined as group IV in the classification of PH [1,2]. It is a progressive disease, resulting from a persistent pulmonary arterial thrombotic obstruction combined with significant peripheral small pulmonary vascular remodeling [3,4,5,6].

The pathophysiology, epidemiology, and risk factors contributing to the development of CTEPH are still widely discussed and not entirely understood [3,6,7,8]. The most important releasing factor is generally accepted to be the occurrence of acute pulmonary embolism with an incomplete thrombus resolution [9,10,11,12,13]. On the other hand, associated small-vessel impairment is often found, which may be (at least partly) explained by secondary over-perfusion and pressure overload of the non-occluded lung areas [7,8,14,15,16,17]. This theory is underlined by the presence of pulmonary vascular changes that show histological similarities with idiopathic pulmonary arterial hypertension (PAH) [1,3,7,8].

**Figure 1:**
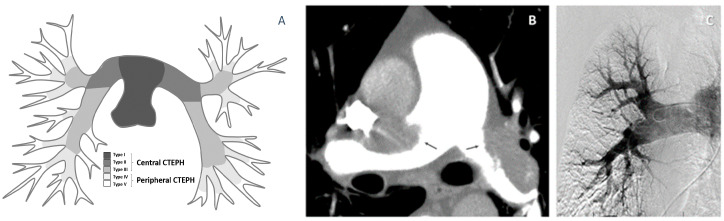
Central vs. peripheral type of CTEPH. (**A**). Scheme of central and peripheral CTEPH, (**B**) central CTEPH (by computer tomography), and (**C**) peripheral CTEPH (by angiography). CTEPH—chronic thromboembolic pulmonary hypertension.

According to the location of pulmonary vascular affection, two major forms of CTEPH (Figure 1A–C) can be distinguished [1,3,5,18,19]. 1. Central CTEPH is characterized by the increase of pulmonary arterial pressure dominantly due to the major vessel thrombotic obstruction. 2. In peripheral CTEPH, the presence of distal vessel thrombotic obstruction (segmental or subsegmental) is usually found. On the other hand, the obstruction extent in the distal form often does not explain the severity of patient’s symptoms; therefore, the combination with diffuse peripheral small pulmonary arteriopathy is assumed.

The differential diagnosis of CTEPH from other forms of PH, as well as distinguishing central from peripheral CTEPH, is crucial for the patient’s further management, interventional options, and overall long-term outcome [2,3,19,20,21].

CTEPH is the only type of PH that offers a potential cure. Surgical pulmonary endarterectomy (PEA) can be performed, provided that the thrombi are located centrally in the main pulmonary artery and main branches or proximally enough to be surgically accessible [22,23,24,25,26].

On the contrary, surgery is difficult to perform and usually is without a patient’s significant clinical improvement if the thrombi are limited to distal parts of the pulmonary arteries. If the occlusion is located at the segmental or subsegmental level, percutaneous balloon pulmonary angioplasty (BPA) can be performed to release the stenotic affection [27,28,29].

If peripheral vascular remodeling is the dominant underlying cause of CTEPH, very frequently no successful surgical or percutaneous intervention is possible to perform, or significant postsurgical clinical symptoms and/or residual pulmonary hypertension is present [26,30]. However, in these patients, the specific medical treatment of pulmonary arterial hypertension might be helpful [30,31,32,33,34].

## 2. Aim of the Study

In our study, patients with CTEPH were analyzed and compared CENTRAL and PERIPHERAL forms of CTEPH in terms of (i) clinical parameters, (ii) echocardiographic parameters, (iii) invasive hemodynamic parameters, and (iv) history of risk factors.

## 3. Patients and Methods

The study included 81 patients (51 male/30 female) with confirmed CTEPH at our institution, where complete evaluation was possible to access. A retrospective study was performed, which obtained data from patients´ medical records at the time of CTEPH diagnosis.

According to the CTEPH lesion locations (as defined by computer tomography scan and/or conventional angiography) were differentiated: 51 patients (63%) with central CTEPH (Group 1—CENTRAL) and 30 patients (37%) with peripheral CTEPH (Group 2—PERIPHERAL).

The following parameters were analyzed and compared in both groups:

- The functional status of the patients defined by functional class (FC-WHO), 6-min walk test (6MWT), and laboratory heart failure assessment with N-terminal pro-brain natriuretic peptide (NTproBNP);
- The echocardiographic parameters acquired by transthoracic echocardiography (TTE): the severity of PAH estimated by the tricuspid regurgitation peak gradient (TR PG) and the left ventricular eccentricity index (LV IE), right-ventricular (RV) dilatation by long-axis diastolic diameter (RVd) measurement, and RV function by the assessment of tricuspid annular plane systolic excursion (TAPSE) and fractional area change (FAC);
- Invasive hemodynamic parameters obtained by right heart catheterization: mPAP, transpulmonary gradient (TPG), cardiac index (CI), and pulmonary vascular resistance (PVR). The severity of PAH according to the mean pulmonary artery pressure (mPAP) was defined as mild (mPAP < 35 mmHg), moderate (mPAP 35–45 mmHg), and severe (mPAP > 45 mmHg);
- The presence of risk factors—acute pulmonary embolism (APE); deep venous thrombosis; congenital thrombophilia; blood type other than “0”; surgery and/or immobilization; thyreopathy; and other autoimmune diseases, such as Crohn disease or ulcerous colitis, pacemaker implantation, splenectomy and a history of malignity was obtained from patients´ medical history.

### Statistical Analysis

The student *t*-test or the one-way analysis of variance test for normally distributed data, and the Wilcoxon test for non-parametric data (age, 6MWT, NTproBNP, echocardiographic and hemodynamic parameters), were employed. In the case of nominal data (gender, NYHA, PAH severity, low CI, and the presence of risk factors), contingence tables were used. Comparisons and logistic regression analyses were performed using JMP version 5.0.1 software (SAS Institute Inc., Cary, NC, USA) and Windows Microsoft Excel 2007. The results were expressed as median and variations for continuous variables and as number and percentage for categorical variables. Univariate analysis was performed, and the differences were considered statistically significant at a significance level of *p* < 0.05.

## 4. Results

In Group 1 (CENTRAL), there were 37 male (72.5%) and 14 female (27.5%) patients, with median age 59 years (25–85 years), compared to Group 2 (PERIPHERAL) with 14 male (46.7%) and 16 female (53.3%), with a median age of 64 years (26–75 years) at the time of diagnosis. A significant difference between Group 1 and Group 2 has been determined in terms of gender (*p* = 0.0198) but not of age at the time of diagnosis (Table 1).

### 4.1. Functional Status

Parameters defining patients´ functional status (Table 1) were slightly better in the Group 1 than Group 2 (FC-WHO II/III/IV % of patients—34/6; 0/6% vs. 26.7/70/3.3%, and 6MWT—407 vs. 388 m) but without a significant difference between the groups. The laboratory value of NTproBNT was increased in both groups (1320 vs. 2335 ng/L), though also without significant difference.

### 4.2. Echocardiographic Parameters

Estimated by echocardiography (Table 1), the severity of RV pressure overload measured by TR PG (77.5 vs. 85 mmHg) showed significant PAH in both groups but without statistical difference. There was also no statistical difference in RV diameter (42 vs. 40 mm), nor in the estimation of RV systolic function (TAPSE—18 vs. 19 mm; FAC—40 vs. 42 %) in both groups.

### 4.3. Hemodynamic Parameters

**Figure 2:**
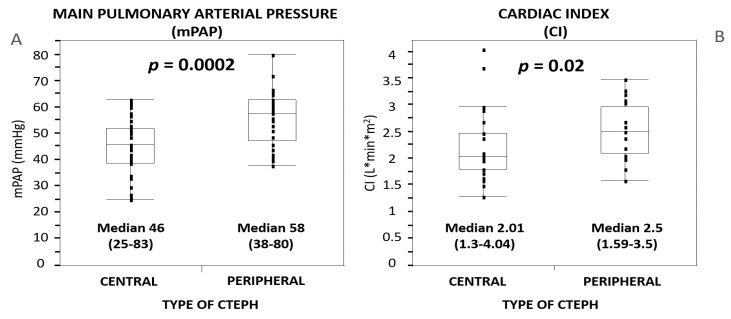
Differences in Group 1 (CENTRAL type) vs. Group 2 (PERIPHERAL type) in hemodynamic parameters: (**A**) mean pulmonary artery pressure (mPAP); (**B**) cardiac index (CI). CTEPH—chronic thromboembolic pulmonary hypertension; CI—cardiac index.

Analyzed hemodynamic parameters were (Table 1), compared to Group 1, significantly higher in Group 2: mPAP (46 vs. 58 mmHg, *p* = 0.0002) (Figure 2A), TPG (34 vs. 47 mmHg, *p* = 0.0005), as well as CI (2.04 vs. 2.5 L·min·m^2^, *p* = 0.02) (Figure 2B). PVR was very high in both groups (9 vs. 10 W.U.), without a statistical difference between groups.

**Figure 3:**
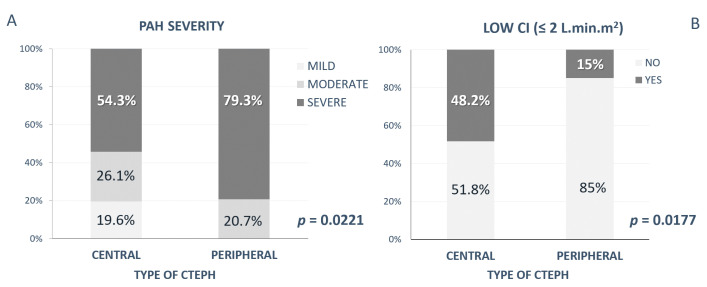
Differences in Group 1 (CENTRAL type) vs. Group 2 (PERIPHERAL type) in: (**A**) invasively assessed PAH severity; (**B**) the presence of low CI (≤2 L.min.m^2^). CTEPH—chronic thromboembolic pulmonary hypertension; CI—cardiac index; and PAH—pulmonary arterial hypertension.

In Group 1, mild/moderate/severe PAH occurred in 9 (19.6 %)/12 (26.1 %)/25 (54.3 %) of patients; in Group 2, mild PAH did not occur, and moderate/severe PAH was present in 6 (20.7 %)/23 (79.3 %) of patients. A comparison of PAH severity in both groups showed a significant difference, with more patients with severe PAH in Group 2 (*p* = 0.0221) (Figure 3A).

Severely decreased CI (defined as CI ≤ 2 L·min·m^2^) was found significantly more frequently in Group 1 (in 13 patients, 48.2 %) than in Group 2 (in 3 patients, 15 %) (*p* = 0.0177) (Figure 3B).

### 4.4. Risk Factors

Traditionally described risk factors were frequently found also in our patients (Table 2.). A high incidence of APE history was found in both groups but significantly was more often present in Group 1 than in Group 2 (93.8% vs. 60%, *p* = 0.0002). Recurrent APE was also determined commonly in both groups (31.3% vs. 26.7%), though with no significant differences. Previous deep venous thrombosis was observed very often in both groups, and although it was observed more often in Group 1 (66.7% vs. 55.2%), there was no statistical significance between both groups. Congenital thrombophilia was present frequently in both groups (37% vs. 34.8%), with no difference.

The presence of a different blood type than “0” (88.4% vs. 71.4%) was found regularly in our patients, as well as a history of previous surgery and/or immobilization (64% vs. 69%), although with no significant difference between groups in terms of either of these risk factors.

More seldom was the occurrence of other risk factors, and without any significant differences in both groups: thyreopathy, the presence of other autoimmune disease (such as Crohn disease, ulcerous colitis, etc.), pacemaker implantation, or splenectomy.

Although the history of malignancy was less common in our cohort, it occurred significantly more often in Group 2 (2% vs. 13.3%, *p* = 0.0426).

## 5. Discussion

CTEPH is usually described with clear high proportion in males, at least in the European population, compared to the Japanese population, where CTEPH was shown to be more frequent in females [3,6,35,36,37]. Interestingly, in our study a strong male predominance was the case only in the CENTRAL form of CTEPH (72.5%), contrary to the PERIPHERAL form of CTEPH, where gender distribution was slightly in favor of females (53.3%) but still did not reach the high frequency of the Japanese cohort. The age distribution in our study included all age groups and did not show a significant difference between the CENTRAL from and the PERIPHERAL form of CTPEH.

Traditionally described risk factors [3,6,7,8,9,10,36,37,38] associated with CTEPH were frequently found also in our patients, though most of them did not differ in both groups.

The only difference was found in the occurrence of APE in patients’ medical history. The history of APE is usually described in up to 75% of cases in the European population but only in about 15–30% of patients in Japan [3,6,35,36,37]. APE in our cohort was commonly present in both groups but significantly more frequently in the CENTRAL form of CTEPH (up to 93.8%) than in patients with the PERIPHERAL form of CTEPH, where in was found “only” in 60%. This may indicate a somewhat different pathophysiological response in both forms. In the CENTRAL form of CTEPH, the standardly described non-resolution of emboli after APE seems to be the dominant underlying factor leading to CTEPH, contrary to the PERIPHERAL form, where other associated risk factors or pathophysiological processes may play an important role as well. This may be underlined by a higher history of malignancy in the PERIPHERAL form of CTEPH (in 13.3%) found in our patients (compared to the CENTRAL form of CTEPH with only 2%), where chronic and/or repeated microembolization or other factors associated with the disease may possibly contribute to this form of CTEPH. Though this interpretation may be limited due to a relatively low number of patients with this combination of diseases.

In our study, the analyses of functional status, 6MWT, or NTproBNP, as well as echocardiography, all pointed to serious clinical impairment and severe PAH but were not able to show differences between the two groups, although the difficulty in precisely analyzing all of the right-ventricular morphological and functional features with standard echocardiographic methods may limit the results.

In our study, invasive hemodynamic evaluation clearly showed a different picture in the CENTRAL form versus the PERIPHERAL form of CTEPH. Although in both groups severe PAH with severely increased pulmonary pressure and high vascular resistance was present, in patients with the PERIPHERAL form of CTEPH the pressure in the pulmonary arterial system was significantly higher. In this group, as many as 79.3% of patients were classified with severe PAH (mPAP > 45 mmHg), whereas in the CENTRAL form of CTEPH, those with severe PAH presented only 20.7% of patients. On the other hand, despite more severe PAH, in patients with the PERIPHERAL form of CTEPH better CI was found, with only 15% of patients with very low CI (≤ 2 L·min·m^2^); compared to the CENTRAL form of CTEPH, where almost in half of the patients severely impaired hemodynamics was present.

The degree of hemodynamic impairment and right-ventricular failure in chronic pulmonary pressure overload is a complex problem and is still not entirely understood. Our findings may have more possible explanations. This may indicate a different pathophysiological response in the right ventricular to pulmonary artery coupling with worse hemodynamic tolerance when the obstruction is located proximally [39,40]. Another explanation could be that the CENTRAL form of CTEPH becomes clinically manifest and is diagnosed sooner, whereas the PERIPHERAL form of CTEPH develops during a longer period of time, with functional compensatory mechanisms present, which enable it to tolerate more severe PAH and still preserve satisfactory cardiac output. This is a well-established phenomenon e.g. in congenital heart defects (especially Eisenmenger syndrome), where the right ventricle is able to tolerate much higher, even suprasystemic pulmonary arterial pressure. This may be partly due to a longer time period of PAH development, enabling a better adaptation model for the right ventricle [41,42,43,44]. In CTEPH the pathophysiological response is though most probably multifactorial.

CTEPH is often defined as a two-compartment pulmonary vascular bed. Distinguishing between CENTRAL and PERIPHERAL affection is crucial in the adjustment of optimal management pathways (PEA and/or BPA versus medical treatment). Despite the clear definition of CTEPH according to imaging tools, there is still quite a lot of clinical and hemodynamic overlapping features between CENTRAL and PERIPHERAL forms, leading to an inadequate response to surgery and residual PAH, which can be found in about 30% of patients after intervention [3]. According to our study, we therefore believe that it is very important not only to describe the presence of CTEPH but also to improve the understanding of the pathophysiology, hemodynamic picture, and associated factors of the disease, especially taking into consideration the possible presence and the degree of peripheral affection of the pulmonary circulation in every patient with CTEPH.

## 6. Study Limitations

The investigation is limited due to the retrospective study design, with limited options to analyze the broad spectrum of possible risk factors and with right-ventricular measurements restricted only to the parameters accessed by standard echocardiographic examination. The results may be also statistically weakened by a smaller number of patients in group 2, and with no possibility to perform multivariate analysis.

## 7. Conclusions

Our study showed significant hemodynamic differences between the CENTRAL and PERIPHERAL forms of CTEPH, with worse hemodynamic parameters found in the CENTRAL form of CTEPH and some differences in the occurrence of risk factors. This may indicate a different pathophysiological (right-ventricular) response in the CENTRAL versus the PERIPHERAL form of CTPEH and/or possible additional influences contributing especially to peripheral pulmonary bed affection. However, these study data need further confirmation. On the other hand, understanding the pathophysiology of CTEPH more deeply and differentiating between various forms of CTEPH may help to implement more targeted and effective therapeutic strategies or to reveal the expected response to treatment options.

## Figures and Tables

**Table 1:** Clinical and hemodynamic characteristics—Group 1 vs. Group 2.

	Gr. 1 (CENTRAL) (*n* = 51)	Gr. 2 (PERIPHERAL) (*n* = 30)	*p* Value
General characteristics			
Age (years)	59 (25–85)	64 (26–75)	0.2
Male gender (%)	72.5	46.7	0.0198
Functional status			
FC-WHO—II/III/IV (% of patients)	34/60/6	26.7/70/3.3	0.64
6MWT (m)	407 (100–650)	388 (120–519)	0.28
NTproBNP (ng/L)	1320 (79.2–8388)	2335 (71.6–8155)	0.1
Echocardiographic parameters			
RVd (mm)	42 (29–58)	40 (32–56)	0.44
TR PG (mmHg)	77.5 (33–126)	85 (55–180)	0.07
FAC (%)	40 (23–65)	42 (29–68)	0.8443
TAPSE (mm)	18 (9–30)	19 (7–29)	0.8829
Invasive hemodynamic parameters			
mPAP (mmHg)	46 (25–83)	58 (38–80)	0.0002
TPG (mmHg)	34 (5–61)	47 (14–62)	0.0005
CI (L·min·m^2^)	2.04 (1.3–4.04)	2.5 (1.59–3.5)	0.02
PVR (W.U.)	9 (1.23–22.2)	10 (3.31–20.5)	0.0621

Data given as median and variance, or %. *p* < 0.05. Gr.—group, FC—functional class, WHO—World Health Organization, 6MWT—6 min walking test, NTproBNP—N-terminal pro-brain natriuretic peptide, RVd—right-ventricular diameter, TR PG—tricuspid regurgitation peak gradient, FAC—fractional area change, TAPSE—tricuspid annular plane systolic excursion, mPAP—mean pulmonary arterial pressure, TPG—transpulmonary gradient, CI—cardiac index, PVR—pulmonary vascular resistance, and W.U.—wood units.

**Table 2:** Risk factors, comparison of Group 1 vs. Group 2.

Risk Factors (*n* = 81)—in %			
	Gr. 1 (Central) (*n* = 51)	Gr. 2 (Peripheral) (*n* = 30)	*p* Value
Acute PE	93.8	60	0.0002
Recurrent acute PE	31.3	26.7	0.66
Deep venous thrombosis	66.7	55.2	0.31
Thrombolytic therapy	10.4	10	0.9529
Congenital thrombophilia	37	34.8	0.86
Blood type other than “0”	88.4	71.4	0.07
History of surgery and/or immobilization	64	69	0.65
Thyreopathy	16	26.7	0.25
Other autoimmune disease (Crohn disease, ulcerous colitis, …)	10	6.7	0.61
Splenectomy	2	3.33	0.71
Pacemaker implantation	4%	0	0.27
Malignancy	2%	13.3%	0.0426

Data given as %. *p* < 0.05. Gr.—group, PE—pulmonary embolism.

## Data Availability

The data underlying this article are available in the article.
